# Pulmonary arterial hypertension: Cellular and molecular changes in the lung

**DOI:** 10.21542/gcsp.2020.3

**Published:** 2020-04-30

**Authors:** Bradley A. Maron

**Affiliations:** Department of Medicine, Division of Cardiovascular Medicine, Brigham and Women’s Hospital, Boston, MA, USA; The Boston VA Healthcare System, West Roxbury, MA, USA

## Abstract

The range of cell types identified in the pathogenesis of pulmonary arterial hypertension (PAH) has expanded substantially since the first pathological descriptions of this disease. This, in turn, has provided needed clarity on the gamut of molecular mechanisms that regulate vascular remodeling and promote characteristic cardiopulmonary hemodynamic changes that define PAH clinically. Insight derived from these scientific advances suggest that the PAH arteriopathy is due to the convergence of numerous molecular mechanisms driving cornerstone endophenotypes, such as plexigenic, hypertrophic, and fibrotic histopathological changes. Interestingly, while some endophenotypes are observed commonly in multiple cell types, such as dysregulated metabolism, other events such as endothelial-mesenchymal transition are cell type-specific. Integrating data from classical PAH vascular cell types with fresh information in pericytes, adventitial fibroblasts, and other PAH contributors recognized more recently has enriched the field with deeper understanding on the molecular basis of this disease. This added complexity, however, also serves as the basis for utilizing novel analytical strategies that emphasize functional signaling pathways when extracting information from big datasets. With these concepts as the backdrop, the current work offers a concise summary of cellular and molecular changes in the lung that drive PAH and may, thus, be important for discovering novel therapeutic targets or applications to clarify PAH onset and disease trajectory.

## Introduction

Among the first pathological descriptions of pulmonary arterial hypertension (PAH) was that of Brinton from Guy’s Hospital (London UK) in 1949.^1^ In that report, an obliterative pulmonary arterial vasculopathy was emphasized, characterized specifically as ‘two separate conglomerations of entangled blood vessels…with complicated aneurysms’. This observation and others^2^ cast the plexiform lesion as a fundamental, distinct, and dramatic feature of pulmonary arterial hypertension (PAH) (previously primary pulmonary hypertension). However, the complex and integrated cellular and molecular underpinnings of vascular remodeling in PAH would only become realized after five decades of comprehensive examination and research.^3,4^

Indeed, the repertoire of cell-types implicated in the pathogenesis of PAH continues to expand, as does understanding of cross-talk mechanisms that permit signaling between cells, as well as cell phenotype switching that has emerged as both a cause and consequence of disease.^5^ This progress has tightened understanding of PAH by aligning disease causes/triggers with intermediate pathophenotypes (endophenotypes) (e.g., fibrosis, cellular proliferation) through a focus on cell type-specific molecular mechanisms. Through this approach, greater insight on integrated pathways that drive PAH pathology has emerged, and, thus, the discovery of new therapeutic targets to improve outcome of patients affected with this highly morbid disease.^6^

An overview of cellular and molecular changes in the PAH lung was presented recently at the Sir Magdi Yacoub Aswan Heart Center Science and Practice Series (Aswan, Egypt), as part of the Pulmonary Vascular Research Institute’s commitment to global education.^7^ The current work discusses topics central to that presentation.

## Dysregulated cellular metabolism: A common endophenotype across various lung cell types

A number of molecular mechanisms in pulmonary artery endothelial cells (PAECs), pulmonary artery smooth muscle cells (PASMCs), and adventitial fibroblasts have been implicated in the pathogenesis of PAH.^8^ In this presentation, a focus on contemporary understanding of metabolism was emphasized.

**Figure 1.**
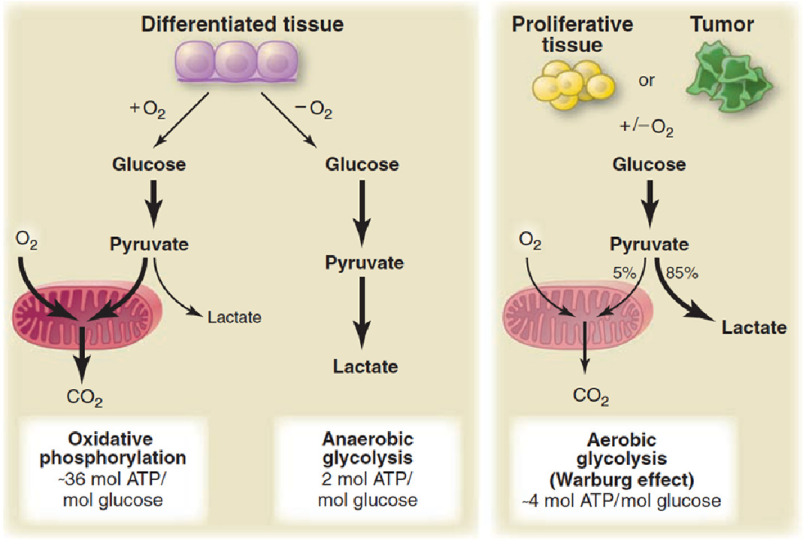
The Warburg phenomenon. Under oxygen rich conditions, oxidative phosphorylation in mitochondria results in efficient adenosine triphosphate (ATP) synthesis. In anaerobic conditions, metabolism shifts in favor of glycolysis, resulting in the preferential synthesis of lactate. The Warburg effect occurs in cancer cells, as well as many vascular cell types in patients with pulmonary arterial hypertension, and is defined by aerobic glycolysis. Redox regulation of hypoxia inducible factor-1 α in the absence of hypoxia is one example of a molecular mechanism that promotes aerobic metabolism, and is important in the development of key PAH endophenotypes, including cellular proliferation and apoptosis-resistance. *Image reproduced with permission from* https://medium.com/@drjasonfung/the-paradox-of-cancers-warburg-effect-7fb572364b8 .

In normal mammalian cells under oxygen-rich conditions, mitochondrial glucose oxidation is the principle metabolic driver of adenosine triphosphate synthesis (ATP). The proliferative pathophenotype of many tumor cells is traced to a predilection for glycolysis rather than mitochondrial glucose oxidation despite ample bioavailable oxygen, resulting in preferential formation of lactate over ATP (Figure 1). This, the Warburg effect, has been proposed as an underlying mechanism by which to explain PASMC proliferation and apoptosis-resistance in PAH.

Building on prior work demonstrating that redox regulation of hypoxia inducible factor (HIF)-1α, initiated via epigenetic mechanism that inhibits anti-oxidant enzyme bioactivity (e.g., superoxide dismutase) to suppress mitochondrial oxidative metabolism,^9^ Marsboom and colleagues emphasized the importance of changes in mitochondrial phenotype in the process of dysregulated metabolism. They observed that human PAH is defined, in part, by a tendency toward mitochondrial fission through cyclin dependent kinase (CDK)-1-dependent phosphorylation of the GTPase Dynamin-related protein-1 at serine-616. This pathway, in turn, was implicated in increased mitotic division and hypertrophic concentric remodeling in plexiform lesions from affected patients.^10^

Genetic predisposition to PAH through a germline mutation in the *BMPR2* gene has long been recognized,^11^ although the mechanistic association between this and cellular metabolism was established recently. The possibility that Bone Morphogenetic Protein Receptor Type 2 (BMPR-2) is important in oxygen-dependent signaling is based, in part, on its regulation of peroxisome proliferator-activated receptor- γ (PPAR- γ)-mediated gene transcription,^12,13^ as well as interplay between BMPR-2 and peroxisome proliferator-activated receptor-gamma coactivator (PGC)-1 α, a transcription factor critical in normal mitochondrial bioenergetic utilization.^14^ Indeed, BMPR2 is shown to control pulmonary endothelial reoxygenation response pathways through pathogenic changes to PGC-1 α in addition to p53, Nuclear factor erythroid 2-related factor 2 (NRF2), and mitochondrial transcription factor A (TFAM).^15^ Such modifications to normal signaling, in turn, lead to a disruption in the membrane potential of mitochondria as well as phenotypic changes in the form of fission and function changes including diminished ATP production.

Like findings in PASMCs and PAECs, adventitial fibroblasts isolated from idiopathic PAH patients and large animal models of hypoxic pulmonary hypertension demonstrate aerobic glycolysis under normoxic cultured conditions.^16^ Similar to other pulmonary vascular cell types, this phenomenon was associated with a perturbation to the normal cellular redox potential, which in adventitial fibroblasts resulted in increased free NADH and the ratio of NADH/NAD^+^.

In a clever series of experiments in control and pulmonary hypertension (PH) adventitial fibroblasts, transcellular flux of radiolabeled glycolytic byproducts, including lactate, was analyzed using high pressure liquid chromatography-mass spectrometry to show increased utilization of glucose for aerobic glycolysis, which was consistent with data from RNA-Seq profiling major differences in the metabolomic signature between cells. The link between dysregulated metabolism and redox imbalance that lead to a proinflammatory cellular phenotype was through co-repressor C-terminal binding protein 1 (CtBP1), as PH adventitial fibroblasts express increased CtBP1 and pharmacological or molecular inhibition of CtBP1 promoted upregulation of genes that promote apoptosis and halt growth.

**Figure 2.**
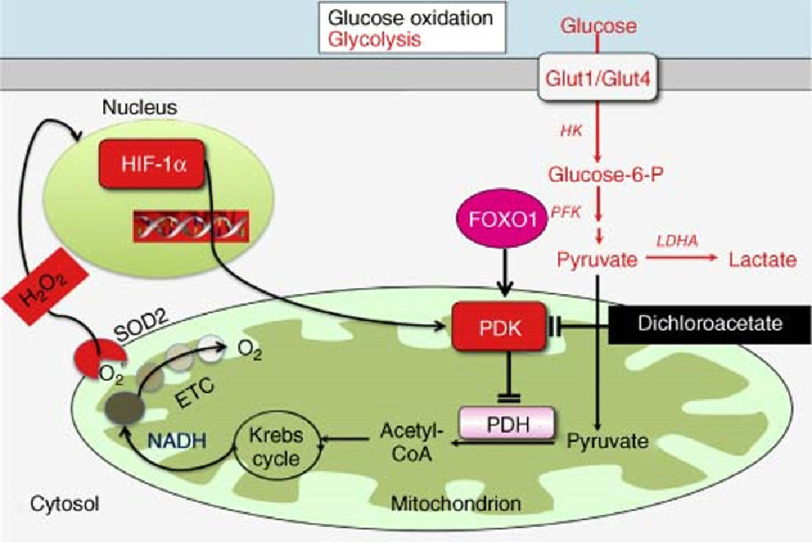
Targeting pyruvate dehydrogenase kinase (PDK) with dichloroacetate for the treatment of pulmonary arterial hypertension. Activation of various transcription factors, including FOXO1, cMyc and HIF-1 α upregulates expression of many glycolytic gene. A common finding in pulmonary arterial hypertension is increased PDK expression, which inhibits PDH and reduces mitochondrial respiration. PDK activation also occurs in the lung in PAH, although the transcriptional regulation and isoform specificity may differ than that seen in the RV. Dichloroacetate inhibits PDK and thereby promotes glucose oxidation and inhibits glycolysis. ETC, electron transport chain; HK, hexokinase; H_2_O_2_, hydrogen peroxide; LDHA, lactate dehydrogenase A; PFK, phosphofructokinase. *Reproduced with permission from Ref.* [37].

Several lines of data implicate the interaction between pyruvate dehydrogenase kinase (PDK) and pyruvate dehydrogenase (PDH) as a critical but modifiable target by which to normalize the metabolic substrate of various pulmonary vascular cell type implicated in PAH (Figure 2).^17,18^ Under normal conditions, PDH catalyzes the conversion of pyruvate to Acetyl-CoA in a reaction that is inhibited by activation of PDK resulting in the formation of a PDH-PDK complex. Importantly, PDK may be induced selectively by activation of HIF-1 α and PDH may be inhibited by inhibition of SIRT3, both of which are biochemical events that define PAH.^19^ These collective observations and other related lines of empiric investigations *in cellulo* and *in vivo* gave rise to the hypothesis that dichloroacetate (DCA), which inhibits PDK (and, thus, removes negative inhibition of PDH by PDK), may be useful clinically.

Michelakis and colleagues completed a 4 month DCA dose-finding (3, 6.25, or 12.5 mg/kg twice daily) clinical trial in PAH patients on disease-specific therapy of <6 month duration.^20^ A total of N=20 patients were enrolled in the study, and reversible peripheral neuropathy was observed in each of the N=5 patients that escalated to the 12.5 mg/kg dose, resulting in discontinuation from the study for N=4 patients. Overall, significant changes were observed in mean pulmonary artery pressure, pulmonary vascular resistance, and 6-minute walk distance post-DCA compared with pre-DCA by a mean of four mmHg, 70 dynes⋅s⋅cm^−5^, and 25 m, respectively. Clinical response was variable within the study cohort, however, and a super responder subgroup emerged. By contrast, a *SIRT3* variant was associated with DCA resistance, potentially due to genetic predisposition to SIRT3-mediated inhibition of PDH in those patients.

**Figure 3.**
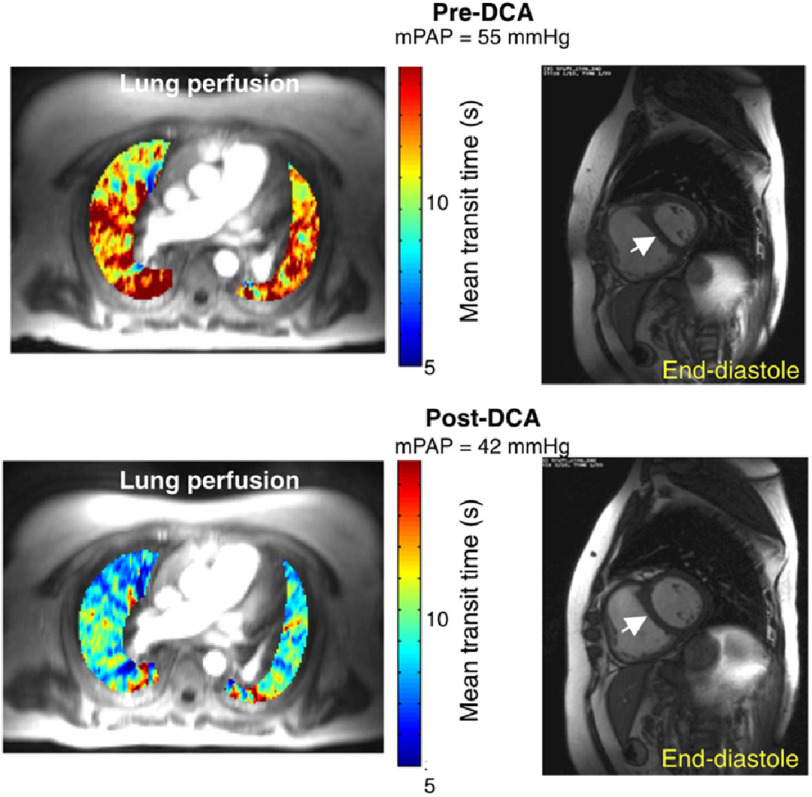
Functional imaging biomarkers in two DCA responders. Magnetic resonance showing gadolinium transit time before and after DCA and resolution of the D-shaped septum (arrow) in the heart of a study patient. Note the color scale of the gadolinium: blue indicates short transit time and increased perfusion; yellow/red indicates a long transit time and little/no perfusion at baseline. *Reproduced with permission from Ref.* [20].

Beyond the important advance of this trial relative to DCA in the context of personalized medicine, the investigators also emphasized novel functional measures that had been generally absent from PAH clinical trials. In particular, MRI images were used to quantify gadolinium transit time across the pulmonary vasculature pre- and post- DCA, and were correlated with morphological changes in the interventricular septum, providing an important measure linking bioactivity with physiology to confirm that changes in hemodynamics were likely due to improved pulmonary arterial flow (Figure 3).

## Pulmonary pericytes

Pulmonary pericytes play an important role in stabilizing endothelial cells, modulating paracrine signaling, and regulating vascular tone through effects on cellular remodeling and immunogenic signaling.^21^ Although generally overlooked in most PAH pathological descriptions, the importance of pericytes has come into focus more recently. In a landmark study that aimed to profile pericyte density and phenotype in PAH, investigators identified a 2-fold increase in pericyte quantity in distal PAH pulmonary arterioles compared to controls and that migration/proliferation of cells could be induced using conditioned medium from cultured PAH PAECs.^22^ Indeed, migration and proliferation could be modulated by enhanced by fibroblast growth factor-2 and interleukin-6, which are two PAH-relevant cytokines.^22^ Findings from this work also shed light on the possibility that smooth muscle fiber formation in distal pulmonary arterioles hinges, in part, on pericyte phenotype switching. This is important, as hypercontractility is a central endophenotype in selected PAH patients and medial thickening is a cornerstone histopathological feature in nearly all PAH patients.

## Endothelial mesenchymal transition

Effacement of pulmonary arterioles in PAH involves plexigenic, fibrotic and hypertrophic vascular remodeling, all of which are processes characterized by the infiltration of cells expressing α-smooth muscle actin (α-SMA). Immunocytochemistry of distal arterioles shows that a plexus network is often observed in PAH lesions. This suggests an integrated process characterized by cells of widely variable lineage, thereby raising speculation that specific biological process may promotes migration toward the inner layer of blood vessels.^23^ Endothelial-to-mesenchymal transition (EndMT) is a change in endothelial cell phenotype to myofibroblast, which permits their translocation locally. Confocal microscopic images from control pulmonary arterioles show a single thin layer of PAECs expressing CD34, CD31, and VE-cadherin aligned parallel to α-SMA-expressing PASMCs. Findings in PAH diverge from these observations: co-expression of CD31 with α-SMA is common in the endothelial, layer as well as the neointima.^24^ Other key markers of EndMT have also been observed in PAH lungs, including Twist-1 and p120-catenin, indicating that evidence of intermediate phases of this process are detectable even in end-stage disease. The functional importance of EndMT is supported by empiric data from rodent PAH models, in which treatment with rapamycin improved key metrics in vascular remodeling that correlated with a reduction in EndMT markers, particularly Twist-1.

## Pulmonary endothelial fibrotic mechanisms

Master switch theories implicate a single molecule as the key driver underlying a given endophenotype. For example, transforming growth factor (TGF)- β is proposed to regulate collagen deposition across different cell- and tissue-types.^25^ However, master switch theories often overlook biofunctionality; in the case of fibrosis, both TGF - β-ddependent adaptive and pathogenic collagen deposition are reported. Applying unbiased analytical strategies that account for overlapping molecular signaling pathways and aim to decipher disease-specific mechanism may provide useful for understanding the pathobiology of fibrotic arterial remodeling in PAH.^26^ Network medicine is well-suited to address this problem, as this approach emphasizes functional or physical relationships between biological components (e.g., proteins) to identify pathways that control or regulate disease processes.^27,28^

We used network medicine to discover the malignancy protein NEDD9 as a previously unrecognized pro-fibrotic protein that is important in pulmonary arterial remodeling.^29^ To accomplish this end, fibrosis genes were collected from various valid resources and defined as either adaptive of pathogenic based on their association with dermal or vascular fibrosis, respectively. After removing genes associated with lung fibrosis *per se* (to avoid including genes implicated in non-vascular forms of pulmonary fibrotic diseases such as idiopathic pulmonary fibrosis), the remaining genes were cross-referenced with array data from vascular endothelial cells treated with the aldosterone, selected because this is a profibrotic hormone observed at increased concentrations in PAH. The resulting gene list was mapped to the human interactome,^30^ which contains information on >14,000 protein-protein interactions, and betweenness central analysis identified NEDD9 as an important network node in the transition from adaptive to pathogenic fibrotic phenotypes.

**Figure 4.**
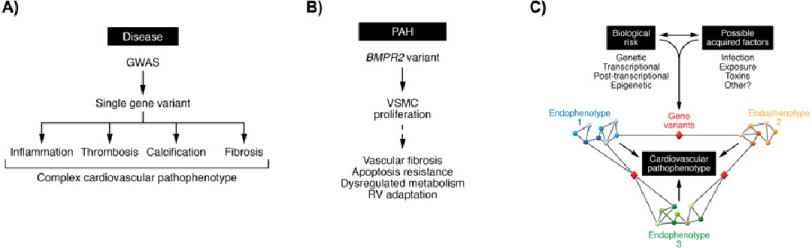
Leveraging a model of convergence to clarify the pathogenesis of pulmonary arterial hypertension. (A) Pulmonary arterial hypertension (PAH) is a complex clinical phenotype that involves many different endophenotypes (e.g., inflammation, thrombosis, fibrosis) that cannot be explained solely by a single pathogenic variant. (B) Heterogeneity in PAH is evident as shown by the relationships among genetic variants (genotypes), the biochemical and cellular consequences of harboring these variants (endophenotypes), and the clinically observed pathophenotype. (C) In a model based on big data and network analyses, specific endophenotypes are determined by modules or a (sub)network of protein-protein interactions within a larger disease network. Crosstalk between pathways that regulate different endophenotypes via a critical gene may occur. In this way, post-transcriptional and epigenetic mechanisms that are important in the pathogenesis of PAH endophenotypes are emphasized and, collectively, converge to produce the complex PAH pathophenotype. *Adapted with permission from Refs.* [38,39].

A functionally critical cysteine at position 18 (Cys^18^) of NEDD9 emerged as a novel redox switch to regulate protein-protein interactions between NEDD9 and SMAD3. Under conditions of oxidant stress akin to PAH, NEDD9 accumulation was observed in PAECs owing to disruption in NEDD9-SMAD3 complex formation, which is required for normal proteasomal degradation of NEDD9. Increased bioavailable NEDD9, in turn, interacts with the cardiac developmental transcription factor NKx2-5^31^ to directly upregulate *COL3A1* transcription, increase collagen III expression, and induce cellular stiffening in PAECs. Furthermore, increased NEDD9 was implicated in paracrine cross-talk via exosome signaling between PAECs and other cell types with fibrotic potential, suggesting that PAECs may function as a portal of entry into a wider cellular fibrotic program.^32,33^

## Conclusions

The portfolio of mechanisms implicated in the pathogenesis of PAH has been expanded greatly in the molecular and big data era, but continues to align tightly with observations made from detailed histopathological analyses. This progress has hinged, in part, on efforts to widen the gamut of cell types involved in vascular remodeling, resulting in several key advances clarifying understanding of dysregulated metabolism, phenotype switching, post-translational events, and cellular cross-talk on key aspects of the PAH arteriopathy. When considered as a collective, overlap and integration between signaling pathways in PAH is recognized as increasingly complex, and less likely to be driven solely by a single sentinel event (Figure 4).^34^ Indeed, emerging research on nutritional, pollution, and other acquired contributors to PAH are steering the field toward better models for predicting incident PAH.^35,36^ Only in this way can knowledge on the convergence of genetic, molecular, and environmental factors be utilized to individualize pathobiology-phenotype in patients. This progress is within the larger mission in the field toward a precision medicine approach for drug development and clinical trial design.

## CONFLICTS OF INTEREST

Dr. Maron is a consultant for Actelion Pharmaceuticals, and is an inventor on a US patent 9,605,047 and US provisional patent applications 24622 and 61/99,754.
